# ChatGPT in Public Cardiovascular Healthcare: Accuracy, Limitations, and Implications

**DOI:** 10.1111/jep.70465

**Published:** 2026-05-03

**Authors:** Lara Borja Mialarett, Thiago Lott Bezerra, Manuela Carneiro Lopes, Juliano Moreira Reis Filho, Julliane V. Joviano‐Santos

**Affiliations:** ^1^ Faculdade de Ciências Médicas de Minas Gerais Belo Horizonte Brasil; ^2^ Post‐Graduate Program in Health Sciences Faculdade de Ciências Médicas de Minas Gerais Belo Horizonte Brasil; ^3^ Laboratório de Investigações NeuroCardíacas Ciências Médicas de Minas Gerais (LINC CMMG) Belo Horizonte Brasil

**Keywords:** artificial intelligence, cardiovascular health, ChatGPT, healthcare delivery

## Abstract

**Introduction:**

ChatGPT is a cost‐effective artificial intelligence (AI) tool designed to facilitate virtual interactions with humans, and its application in healthcare is expanding. However, research on ChatGPT's effectiveness in public healthcare, particularly for cardiac patients, is still limited. This study aims to evaluate ChatGPT's potential in managing cardiovascular health for patients with acute or chronic cardiac conditions.

**Methods:**

We analyzed real medical records from ‘The Cardiovascular Care’ program, affiliated with a university outpatient clinic. ChatGPT's performance was evaluated in terms of its ability to analyze clinical cases, propose diagnoses, and recommend appropriate actions. We also assessed whether ChatGPT's accuracy and errors varied depending on disease severity, rarity, mortality risk, and urgency.

**Results:**

When compared to physicians' records, ChatGPT provided correct responses in 43% of diagnostic hypotheses, 5% of recommended supplementary exams, and 10% of laboratory tests. It showed significant accuracy and discernment in diagnosing conditions influenced by factors such as severity, rarity, risk of death, and urgency. However, this discernment did not extend to recommendations for supplementary exams and laboratory tests. Interestingly, while ChatGPT's responses in these areas were often only partially accurate, they tended to be more detailed, sometimes unnecessarily so, than those provided by physicians. Diagnostic hypotheses from multiple models, including ChatGPT Health, DeepSeek, Gemini Pro, Perplexity AI, and ESC Chat, were also evaluated. Performance varied across models, with ChatGPT demonstrating the highest diagnostic accuracy among those assessed, despite still producing incorrect outputs.

**Conclusion:**

Although ChatGPT demonstrates some diagnostic capability, its overall reliability remains questionable, with performance at times approaching random chance. Caution is advised when considering its use in clinical decision‐making.

## 1 Introduction

Artificial Intelligence (AI) is a rapidly evolving technology that enables machines to simulate aspects of human intelligence, including reasoning, learning, and problem‐solving [1]. Among contemporary AI systems, the Chat Generative Pre‐trained Transformer (ChatGPT) is a Large Language Model (LLM) capable of generating context‐sensitive text through probabilistic pattern recognition derived from extensive training on large corpora of human language data [2]. Unlike rule‐based or explicitly programmed diagnostic systems, conventional LLMs do not reason through pathophysiological mechanisms; rather, they generate outputs based on statistical associations between textual patterns. This distinction raises important questions regarding the nature of AI‐generated clinical suggestions and their relationship to professional medical judgment.

In the medical field, ChatGPT has been explored for applications including patient care support, medical education, initial clinical screening, research assistance, and integration with healthcare systems [3, 4, 5, 6]. Its potential in medical education was initially demonstrated in studies evaluating its performance on the United States Medical Licensing Examination (USMLE) [7, 8]. However, performance on standardized examinations does not necessarily translate into reliability within real‐world clinical environments, where ambiguity, multimorbidity, contextual variability, and time‐sensitive decision‐making are integral components of practice. Furthermore, despite its promise, ChatGPT is not without limitations, including inherent algorithmic biases and ethical concerns related to its use in healthcare settings [9, 10, 11]. These concerns underscore the need for systematic evaluation of its outputs within authentic clinical contexts.

Despite the growing presence of AI in healthcare, there remains limited research validating ChatGPT as a practical tool in routine clinical care, particularly in cardiovascular medicine. Cardiovascular diseases (CVDs) are responsible for approximately 17.8 million deaths annually, representing a major contributor to global mortality and healthcare burden [12, 13]. In addition to their high mortality, CVDs require continuous monitoring, early diagnosis, and complex clinical decision‐making, placing substantial pressure on health systems. Public healthcare systems, particularly those serving underserved populations, often face significant resource limitations while managing large patient volumes and high clinical complexity. This scenario is especially relevant in cardiovascular care, where timely diagnostic reasoning and appropriate test selection are essential to prevent adverse outcomes. In such contexts, scalable and low‐cost technologies, including LLMs, may offer potential support for clinical workflows.

Importantly, clinical characteristics such as disease severity, urgency, mortality risk, symptom specificity, and comorbidity burden may substantially influence the responses generated by ChatGPT [14]. Because LLMs operate through probabilistic patterns [2], presentations with classical and well‐defined features (e.g., typical chest pain or decompensated heart failure) may be more likely to elicit guideline‐consistent outputs. In contrast, complex cases involving atypical symptoms, overlapping syndromes, or multiple comorbidities may increase interpretive ambiguity, potentially affecting prioritization of differential diagnoses and recommendations for extra examinations or laboratory tests investigations. Variability in clinical presentation might therefore directly impact the consistency, depth, and clinical alignment of AI‐generated responses, highlighting the importance of contextual framing and continued human oversight [15].

In this context, we hypothesize that ChatGPT, as an accessible LLM tool, may generate diagnostic hypotheses and recommendations for complementary examinations comparable to those documented by physicians in clinical practice. This study aims to assess whether ChatGPT's diagnostic hypotheses align with those recorded in medical charts and whether its recommendations for complementary examinations and laboratory tests are clinically coherent. Additionally, we evaluate whether its performance varies according to disease severity, rarity, mortality risk, and clinical urgency. Finally, we compare ChatGPT's diagnostic hypotheses with those generated by other emerging LLM models.

## 2 Methods

### 2.1 Study Design and Ethical Aspects

Our study was approved by the Research Ethics Committee (#67119822.9.0000.5134) and conducted in accordance with the World Medical Association's Code of Ethics (Declaration of Helsinki) for human research. We evaluated the competence of ChatGPT‐3.5 in analyzing, diagnosing, and recommending appropriate actions based on clinical case data from ‘The Cardiovascular Care’, within an outpatient clinic at the Hospital of Medical Sciences of Minas Gerais, Brazil. The assessment considered various factors, including patient gender, age, and clinical history. Each stage of the clinical workflow, from generating diagnostic hypotheses to recommending complementary exams and laboratory tests, was systematically analyzed and evaluated.

### 2.2 Protocol Study Procedures

We analyzed 168 medical records from patients who accessed public healthcare services for initial and follow‐up consultations. After excluding 68 records due to insufficient information, we successfully collected data from 100 cardiovascular cases. We then conducted research using ChatGPT, formulating questions based on factors such as gender, age, risk factors, and the primary complaint documented in each medical record. These questions aimed to assess primary diagnostic hypotheses, recommended complementary exams, and suggested laboratory tests, all tailored to each patient's specific condition. The prompts were intentionally designed to simulate the real‐world sequential clinical reasoning in which complementary examinations and suggested laboratory tests should be selected based on an initial diagnostic hypothesis. Data was sourced from electronic health records and extracted from key sections, including medical history, physical examinations, progress notes, prescriptions for supplementary exams, and laboratory test results. The extracted text was entered verbatim into ChatGPT. Responses generated by ChatGPT were evaluated by certified cardiologists with extensive experience in the cardiovascular field, who assessed the accuracy and reliability of its recommendations.

To further explore variability in diagnostic hypotheses generation, the same clinical prompts were submitted to other LLMs, including ChatGPT Health, DeepSeek, Gemini Pro, Perplexity AI, and ESC Chat.

### 2.3 Statistical Analyses

Descriptive statistics were used to summarize the main characteristics of the sample, and variables were presented as absolute frequencies and percentages. Associations between categorical variables and ChatGPT performance across the evaluated domains were assessed using Fisher's exact test for r × c contingency tables, due to the presence of small expected cell counts in some categories. The strength of association between variables was estimated using Cramer's V. All statistical analyses were performed using R (version 4.3.2), and p‐values were considered statistically significant at the 0.05 level.

## 3 Results

The analysis was structured into three predefined domains: (1) diagnostic hypotheses, (2) recommendations for supplementary exams, and (3) laboratory test requests. To ensure consistency, the prompts posed to ChatGPT followed a structured format, as outlined in the Supplementary Tables (1–3). ChatGPT's responses were then compared with the medical records, and its accuracy was evaluated using the following criteria: Yes (100% accuracy), No (0% accuracy), or Partially (50% accuracy).

### 3.1 ChatGPT and Diagnostic Hypothesis

**Figure 1:**
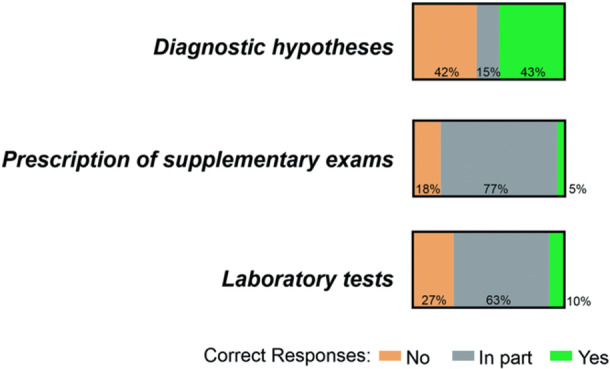
Conjoint Domain Analysis. Examination of three domains: diagnostic hypothesis, prescription of supplementary exams, and laboratory tests.

We hypothesized that ChatGPT could serve as an adjunctive tool in public healthcare, particularly within ‘The Cardiovascular Care’ program. To test this, we first examined the concordance between ChatGPT's diagnostic hypotheses and those recorded by clinicians. After posing standardized questions to the AI, we compared its responses with medical records, observing 43% accuracy, 42% errors, and 15% partial matches (Figure 1). In this domain, ChatGPT provided only a single diagnostic hypothesis for each clinical scenario. A response was considered correct only when there was 100% agreement between ChatGPT's output and the diagnosis recorded in the medical file.

Next, we explored potential associations between ChatGPT's accuracy and clinical factors, including disease severity, rarity, risk of death, and urgency of care. These classifications were determined by an experienced cardiologist. Within our sample, 65% of cases were categorized as severe, none were classified as rare (100% were common), 59% carried a risk of death, and 50% required urgent medical attention (Table 1).

**Table 1:** Patients' conditions assessed by an experienced cardiologist.

Features of the examined conditions as assessed by the cardiologist	*N* = 100^a^
Severity?	
No	35 (35%)
Yes	65 (65%)
Rarity?	
No	100 (100%)
Risk of death?	
No	41 (41%)
Yes	59 (59%)
Urgency of patient attention?	
No	50 (50%)
Yes	50 (50%)
Gender	
Female	48 (48%)
Male	52 (52%)

^a^ *n* (%)

We then conducted an association analysis between ChatGPT's performance and these clinical variables. As shown in Table 2, a significant association was found between ChatGPT's success rate and disease severity, suggesting that ChatGPT performed better in diagnosing more severe cases (*p* < 0.001/Cramer's V = 0.45). Additionally, significant associations were observed between ChatGPT's accuracy and both risk of death (*p* < 0.001/Cramer's V = 0.44) and urgency of care (*p* = 0.011/Cramer's V = 0.27), indicating that ChatGPT was more likely to provide correct diagnoses when these factors were present.

**Table 2:** Examining ChatGPT performance in the diagnostic hypothesis domain: accuracy, errors, and partial matches considering different contexts.

Was ChatGPT successful in relation to the diagnostic hypothesis?					
Features	Yes, *N* = 43	Partially, *N* = 15	No, *N* = 42	Total, *N* = 100	*p* value*/Cramer's V
Severity?					< 0.001/0.45
No	10 (23%)	0 (0%)	25 (60%)	35 (35%)	
Yes	33 (77%)	15 (100%)	17 (40%)	65 (65%)	
Rarity?					
No	43 (100%)	15 (100%)	42 (100%)	100 (100%)	
Risk of death?					< 0.001/0.44
No	14 (33%)	0 (0%)	27 (64%)	41 (41%)	
Yes	29 (67%)	15 (100%)	15 (36%)	59 (59%)	
Urgency of patient attention?					0.011/0.27
No	18 (42%)	4 (27%)	28 (67%)	50 (50%)	
Yes	25 (58%)	11 (73%)	14 (33%)	50 (50%)	
Gender					0.054
Female	24 (56%)	3 (20%)	21 (50%)	48 (48%)	
Male	19 (44%)	12 (80%)	21 (50%)	52 (52%)	

*n* (%)

* Fisher's Exact Test.

As a control measure, we examined whether patient gender influenced ChatGPT's accuracy and found no significant difference. Overall, these findings suggest that ChatGPT's ability to generate accurate diagnostic hypotheses is influenced by disease severity, risk of death, and urgency of care but remains unaffected by patient gender.

### 3.2 ChatGPT and Prescription of Supplementary Exams

We investigated whether ChatGPT could recommend supplementary exams in a manner consistent with clinicians' recommendations documented in medical records. The data revealed that ChatGPT's recommendations fully matched those of clinicians in only 5% of cases. In 18% of cases, it failed to suggest the correct supplementary exams, while in 77% of cases, it provided partial recommendations (Figure 1). Notably, although ChatGPT's responses were often only partially correct, they were generally more detailed than those recorded in the medical files. Additionally, we performed an association analysis to examine potential associations between ChatGPT's accuracy in recommending supplementary exams and various clinical conditions. However, no significant associations were found between ChatGPT's success rate and the different clinical scenarios related to complementary exams (Table 3).

**Table 3:** Examining ChatGPT performance in the prescription of supplementary exams domain: accuracy, errors, and partial matches considering different contexts.

Was ChatGPT successful in relation to the prescription of supplementary exams?					
Features	Yes, *N* = 5	Partially, *N* = 77	No, *N* = 18	Total, *N* = 100	*p* value*
Severity?					0.304
No	1 (20%)	25 (32%)	9 (50%)	35 (35%)	
Yes	4 (80%)	52 (68%)	9 (50%)	65 (65%)	
Rarity?					
No	5 (100%)	77 (100%)	18 (100%)	100 (100%)	
Risk of death?					0.309
No	1 (20%)	30 (39%)	10 (56%)	41 (41%)	
Yes	4 (80%)	47 (61%)	8 (44%)	59 (59%)	
Urgency of patient attention?					0.304
No	1 (20%)	38 (49%)	11 (61%)	50 (50%)	
Yes	4 (80%)	39 (51%)	7 (39%)	50 (50%)	
Gender					0.609
Female	3 (60%)	38 (49%)	7 (39%)	48 (48%)	
Male	2 (40%)	39 (51%)	11 (61%)	52 (52%)	

*n* (%)

* Fisher's Exact Test.

### 3.3 ChatGPT and Laboratory Tests

Finally, we evaluated the aspects related to the laboratory tests. In this regard, ChatGPT correctly assessed the examinations in only 10% of patients. In the other 63% of cases, it was partially correct and in 27% of patients, incorrect information was provided, as shown in Figure 1. Again, while ChatGPT's responses were often only partially accurate in this field, they provided more detailed information, sometimes unnecessarily so, compared to the entries in the medical file. Subsequent analysis, considering associations with the previously mentioned scenarios, revealed no significant associations observed between the variables of interest, as presented in Table 4.

**Table 4:** Examining ChatGPT performance in the laboratory tests domain: accuracy, errors, and partial matches considering different contexts.

Was ChatGPT successful in relation to the laboratory tests?					
Features	Yes, *N* = 10	Partially, *N* = 63	No, *N* = 27	Total, *N* = 100	*p* value*
Severity?					0.608
No	5 (50%)	21 (33%)	9 (33%)	35 (35%)	
Yes	5 (50%)	42 (67%)	18 (67%)	65 (65%)	
Rarity?					
No	10 (100%)	63 (100%)	27 (100%)	100 (100%)	
Risk of death?					0.347
No	6 (60%)	26 (41%)	9 (33%)	41 (41%)	
Yes	4 (40%)	37 (59%)	18 (67%)	59 (59%)	
Urgency of patient attention?					0.739
No	6 (60%)	32 (51%)	12 (44%)	50 (50%)	
Yes	4 (40%)	31 (49%)	15 (56%)	50 (50%)	
Gender					0.228
Female	5 (50%)	34 (54%)	9 (33%)	48 (48%)	
Male	5 (50%)	29 (46%)	18 (67%)	52 (52%)	

*n* (%)

* Fisher's Exact Test.

### 3.4 Conjoint Domain Analysis

In summary, Figure 1 depicts the analysis of the three domains studied: diagnostic hypothesis, prescription of supplementary exams, and laboratory tests. The data reveal that 43% of correct responses are associated with the diagnostic hypothesis, 5% with the conduct of complementary exams, and 10% with the laboratory tests. This indicates that the AI performed better when employed for diagnostic hypotheses. Notably, in the last two domains, the partial rates were high at 63% and 77%, respectively. This partial accuracy may pose challenges when utilizing AI for more decisive actions in conducting complementary exams and laboratory tests.

**Figure 2:**
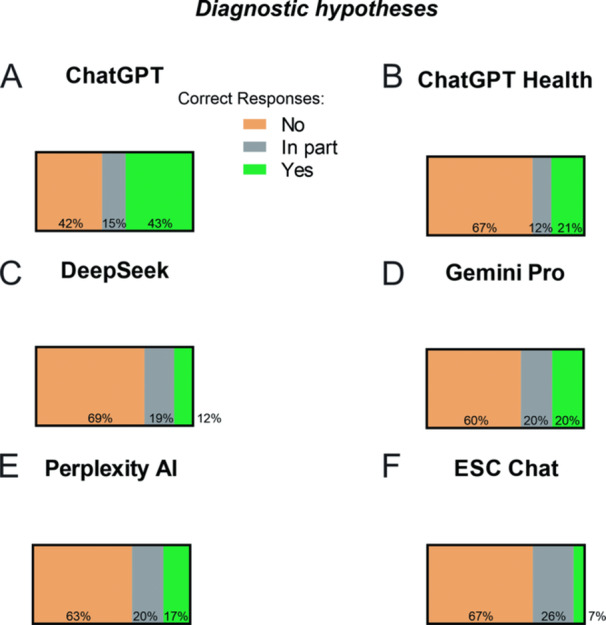
Diagnostic hypotheses generated by different Large Language Models. (A) ChatGPT; (B) ChatGPT Health; (C) DeepSeek; (D) Gemini Pro; (E) Perplexity AI; and (F) ESC Chat. Each panel illustrates the distribution of diagnostic hypothesis classifications generated by the respective model according to the study criteria (correct, partially correct, and incorrect).

Figure 2 and Supplementary Tables (4–8) show the performance of the evaluated models in generating diagnostic hypotheses: ChatGPT (A), ChatGPT Health (B), DeepSeek (C), Gemini Pro (D), Perplexity AI (E), and ESC Chat (F), while Table 5 provides a comparative analysis of ChatGPT and the other evaluated models. In this analysis, responses categorized as “correct” and “partially correct” were combined and defined as “potentially correct.” ChatGPT achieved 58% potentially correct responses and 42% incorrect responses and was used as the reference model. All evaluated models demonstrated significantly higher proportions of incorrect responses compared with ChatGPT, indicating inferior performance in this domain (Table 5).

**Table 5:** Comparing ChatGPT performance against different models in the diagnostic hypothesis domain.

Models	Potentially correct (%)	Incorrect(%)	*p* value*
ChatGPT	58	42	—
ChatGPT Health	33	67	0.0006
DeepSeek	31	69	0.0002
Gemini Pro	40	60	0.0160
Perplexity AI	37	63	0.0045
ESC Chat	33	67	0.0006
(%)			

* Fisher's Exact Test, versus ChatGPT.

## 4 Discussion

Our study found that ChatGPT generated both correct and incorrect diagnostic hypotheses in nearly equal proportions compared to those recorded by clinicians in medical records. This balance between accuracy and error underscores the need for careful evaluation of ChatGPT's reliability in cardiovascular diagnostics while also highlighting areas for potential improvement.

ChatGPT's performance varied according to the clinical complexity, symptom specificity, and comorbidity burden of each case. In relatively straightforward scenarios, such as isolated chest pain in low‐risk individuals or typical hypertensive presentations, the model often aligned with expert clinical decision‐making by recommending guideline‐consistent initial investigations, resulting in correct classifications. However, in cases involving atypical symptoms, overlapping syndromes, or multiple comorbidities, ChatGPT frequently failed to adequately contextualize diagnostic priorities, leading to partially correct or incorrect responses. Notably, the model's generation of diagnostic hypotheses appeared to be influenced by cues related to the perceived severity and urgency of the clinical scenario. Cases suggestive of higher mortality risk tended to prompt different patterns of diagnostic prioritization, indicating that elements of the clinical narrative associated with risk and acuity may shape how the model structures and ranks potential diagnoses.

It is important to observe that ChatGPT's performance declined in recommending supplementary exams, which may be due to inconsistencies in how medical records are documented. Variability in physician documentation styles can hinder AI's ability to analyze data effectively. Additionally, physicians in our study worked within the public healthcare system, which has resource limitations and restricted test availability. In contrast, ChatGPT does not consider these constraints and frequently suggests a comprehensive range of potentially beneficial tests, some of which may be unnecessary or unavailable at the time. In such scenarios, physician judgment remains crucial in filtering and prioritizing medical recommendations. Analysis of supplementary exam requests revealed also substantial variability in ChatGPT's performance across clinical scenarios. Correct classifications were predominantly observed in presentations with clear diagnostic pathways, in which the model appropriately prioritized first line investigations, particularly electrocardiography, transthoracic echocardiography, ambulatory blood pressure monitoring, and Doppler ultrasonography, in close agreement with expert clinical decision making.

In a subset of cases, ChatGPT identified relevant tests but demonstrated limitations in clinical prioritization, frequently omitting essential examinations or recommending redundant and indiscriminate use of advanced imaging modalities. Incorrect classifications were mainly associated with complex or atypical presentations, in which the model proposed diagnostic strategies that diverged from both recorded clinical decisions and guideline‐based practice, including inappropriate test sequencing, failure to request critical evaluations, and overreliance on high complexity imaging without adequate clinical justification. Notably, in several instances, ChatGPT provided a more comprehensive diagnostic workup than that documented in the medical records, reflecting a tendency toward broader and more inclusive testing strategies, sometimes unnecessary.

Regarding medication prescription, a direct comparison between physicians and ChatGPT was not possible, as the AI does not prescribe medications. In all evaluated cases, ChatGPT advised users to consult a physician for pharmacological management, reinforcing the essential role of healthcare professionals and the current impossibility of AI replacing them [16]. It is important to emphasize that each patient is unique, and pharmacological treatment depends on a physician's comprehensive assessment, including the patient's overall health status, medical history, potential drug interactions, and other relevant factors.

LLM technology is still evolving, and its applications in cardiology are expected to expand significantly. AI has the potential to enhance various aspects of cardiovascular healthcare, from advancing scientific knowledge to improving clinical assistance, as highlighted in a recent review on AI in cardiology [17]. Additionally, AI is showing promising applications in diagnostic procedures, such as electrocardiograms and electrophysiology, as well as in imaging techniques, including echocardiography, cardiac MRI, and nuclear medicine [18].

The overall volume of AI‐related publications in medicine has expanded markedly in recent years, with exponential growth in research outputs across multiple medical specialties. Cardiology has emerged as one of the leading fields adopting these technologies, and projections indicate sustained expansion of AI integration in healthcare in the coming years [19]. Studies specifically focused on cardiovascular applications represent a substantial yet proportionally smaller subset, underscoring both the rapid evolution of the discipline and the need for more targeted, domain‐specific validation research [20]. Furthermore, evaluations highlight the increasing scientific and clinical interest in AI‐driven solutions and provide an analytical framework for understanding how these technologies may be systematically incorporated into clinical workflows to improve diagnostic precision, optimize therapeutic strategies, and ultimately enhance patient outcomes [21].

The risk of ChatGPT providing inadequate medical advice in real cases presents significant legal challenges. Key questions arise regarding liability, whether the responsibility for incorrect AI‐generated medical guidance lies with the patient, the treating hospital, or OpenAI. These concerns highlight the urgent need for comprehensive legal frameworks to define accountability in AI‐assisted healthcare [22]. Privacy issues are another critical challenge. The collection, storage, and processing of confidential patient data must adhere to strict privacy regulations to ensure data protection and security. Finally, the risk of unauthorized access or data breaches remains a concern, especially as AI systems interact with both patients and healthcare providers, potentially handling sensitive medical information [23].

Besides, prompt optimization becomes another critical determinant of output quality when using LLMs in biomedical settings, as the clarity and specificity of the input directly shape response accuracy. Designing well‐structured prompts is fundamental to optimizing the effectiveness of ChatGPT, and the continuous refinement of prompt strategies significantly contributes to improving its performance [24]. Formulating precise and detailed questions, especially those addressing specific aspects of clinical cases, facilitates the generation of more focused and meaningful responses.

The diagnostic hypothesis prompts were additionally evaluated using several tools with different architectures, including ChatGPT Health (a novel interface), DeepSeek, Gemini, Perplexity AI, and ESC Chat. ChatGPT Health is a healthcare‐oriented deployment of ChatGPT that incorporates additional medical safety guardrails and guidance for health‐related queries, whereas conventional ChatGPT is a general‐purpose model designed to respond across a wide range of domains [25]. DeepSeek, represents a newer generation of LLMs designed to achieve high reasoning performance through large‐scale training and efficient model architectures, with reported strong performance in complex reasoning and mathematical domains [26]. Gemini, developed by Google, is a multimodal LLM architecture capable of processing and integrating different types of information, including text, images, and structured data [27]. Some models, such as Perplexity AI, incorporate retrieval‐augmented generation mechanisms that dynamically access external information sources to improve factual grounding and provide referenced responses [28]. Finally, ESC Chat represents a domain‐restricted clinical chatbot trained exclusively on European Society of Cardiology clinical practice guidelines, providing responses grounded in structured, evidence‐based cardiovascular recommendations. Because these models differ substantially in training data scope, architectural design, knowledge retrieval strategies, and degree of domain specialization, their diagnostic hypothesis generation also varied, as observed.

An important consideration in interpreting all findings relates to the classification of “partially correct” responses. In this category, the models do not produce entirely incorrect outputs; rather, they often provide relevant elements that may assist in guiding diagnostic reasoning when accompanied by appropriate clinical supervision. In several instances, follow‐up prompting or iterative questioning could lead the models to refine their responses and arrive at a correct diagnosis, suggesting that these outputs represent a potentially valid diagnostic foundation rather than definitive errors. In this study, a 100% accuracy threshold was defined as exact concordance between the models' outputs and the reference clinical records, including variations in wording. Notably, a proportion of responses categorized as “partially correct” could be considered fully correct after minor adjustments in terminology or interpretation, particularly when evaluated within a supervised clinical context. These findings highlight the potential role of such models as supportive tools in clinical reasoning, while underscoring the continued importance of expert oversight.

A synthesis of the cases presented in Supplementary Tables reveals consistent patterns that help distinguish correct from incorrect diagnostic outputs. Correct calls were predominantly associated with classic and well‐structured clinical presentations, particularly when hallmark symptom clusters were present (e.g., exertional chest pain with radiation suggesting angina, or dyspnea with orthopnea and edema indicating heart failure), often combined with compatible cardiovascular risk factors. In contrast, incorrect or partially correct responses tended to occur in symptoms with mixed gastrointestinal, neurological, or systemic features. Additionally, errors were more frequent in cases requiring finer diagnostic discrimination (e.g., differentiating hypertensive disease from structural cardiomyopathies or non‐cardiac causes), suggesting a limitation in weighting less prominent but decisive clinical clues.

It is important to acknowledge some limitations of this study. Detailed and comprehensive documentation is essential for patient care and data retrieval, allowing physicians to track clinical information over time [29, 30]. Since different healthcare professionals contributed to the medical records used in this study, the recorded data varied based on individual documentation styles. Furthermore, incomplete or inconsistently recorded information may have led ChatGPT to incorrect conclusions. Another key limitation is that physicians, through direct patient interactions, have access to additional contextual information not available to ChatGPT. While AI can assist in clinical reasoning, it cannot replace the nuanced decision‐making of a physician who engages directly with the patient.

As already mentioned, the medical records from this study were derived from real cases managed in ‘The Cardiovascular Care’ program and reflected routine clinical practice rather than being generated from a single predefined protocol. Although diagnoses and management decisions were grounded in national and international cardiovascular guidelines applicable at the time of care, and all cases, including classifications of severity, risk of death, and urgency, were independently reviewed by experienced certified cardiologists, the study design was retrospective and observational. Patient outcomes were not longitudinally tracked to externally validate each case as a gold standard. Consequently, physician‐documented medical records were adopted as the reference standard, and the absence of prospective outcome validation represents an inherent methodological limitation. However, our study evaluates ChatGPT using real‐world public cardiovascular care data, providing clinically grounded insights into its strengths, weaknesses, and practical implications within routine healthcare settings.

## 5 Conclusion

In conclusion, this study demonstrates that ChatGPT shows some capability in generating diagnostic hypotheses in cardiovascular clinical scenarios but limited concordance with clinicians in recommending supplementary exams and laboratory tests. The high proportion of partially correct recommendations in complementary exams and laboratory investigations highlights important limitations for clinical decision‐making. Together, these findings suggest that ChatGPT may serve as a supportive tool for diagnostic reasoning but should not replace clinical judgment, emphasizing the need for continued human oversight and further validation before integration into routine healthcare practice.

## Conflicts of Interest

The authors declare no conflicts of interest.

## Supporting information

Supporting Table 1

Supporting Table 2

Supporting Table 3

Supporting Table 4

Supporting Table 5

Supporting Table 6

Supporting Table 7

Supporting Table 8

## Data Availability

The data that support the findings of this study are available from the corresponding author upon reasonable request.
