# Supplementary material for: ChatGPT in Public Cardiovascular Healthcare: Accuracy, Limitations, and Implications
Source: J Eval Clin Pract. 2026 May 3;32:e70465. doi: 10.1111/jep.70465 (PMC13135820; doi:10.1111/jep.70465)
Supplement: Supplementary file 2 — Supporting Table 2 [file JEP-32-0-s005.docx]

**Supplementary Table 2:** Questions designed for ChatGPT were formulated following this model: “For a patient [Man (M) or Woman (W)], aged (xx) with (Risk factors) and presenting (Chief complaint)-as seen in Supplementary Table 1- what supplementary exams should I request?”

| **Reply from ChatGPT** | **Recorded in the medical file** | **Was ChatGPT successful?** |
| --- | --- | --- |
| Electrocardiogram, Exercise stress test, Echocardiogram, Coronary angiography, Myocardial perfusion scintigraphy, 24-hour Holter monitoring | Myocardial perfusion scintigraphy | In part |
| Electrocardiogram, Exercise stress test, Echocardiogram, Chest X-ray, Coronary computed tomography angiography, 24-hour Holter monitoring | Exercise stress test, Computed tomography angiography, Chest CT | In part |
| Electrocardiogram, Exercise stress test, Echocardiogram, Myocardial perfusion scintigraphy, Coronary CT angiography | Ambulatory blood pressure monitoring, Electrocardiogram, Echocardiogram | In part |
| Electrocardiogram, Exercise stress test, Echocardiogram, Chest X-ray, Coronary CT angiography | Exercise stress test, Echocardiogram | In part |
| Electrocardiogram, Echocardiogram, 24-hour Holter monitoring | 24-hour Holter monitoring, Electrocardiogram, Echocardiogram with Doppler | Yes |
| Esophagogastroduodenoscopy, Electrocardiogram, Echocardiogram | Echocardiogram with Doppler, Myocardial perfusion scintigraphy, Digestive endoscopy | In part |
| Electrocardiogram, Echocardiogram, Chest X-ray | 24-hour Holter monitoring, Two-dimensional echocardiogram with Doppler | In part |
| Electrocardiogram, Exercise stress test, Myocardial perfusion scintigraphy, Coronary angiography | 24-hour Holter monitoring, Chest X-ray, Electrocardiogram, Myocardial perfusion scintigraphy | In part |
| Electrocardiogram, Exercise stress test, Echocardiogram, Myocardial perfusion scintigraphy, Coronary angiography, Cardiac magnetic resonance imaging | CT scan, Exercise stress test, Myocardial perfusion scintigraphy | In part |
| Coronary angiography, Coronary CT, Cardiac magnetic resonance imaging, Stress echocardiography, Myocardial perfusion scintigraphy, Cardiac PET-CT | Transthoracic echocardiogram with Doppler | No |
| Electrocardiogram, Chest X-ray, Echocardiogram, Exercise stress test, Coronary angiography, Cardiac magnetic resonance imaging | Exercise stress test | In part |
| Electrocardiogram, Echocardiogram, Spirometry, Chest X-ray, CT scan | Myocardial perfusion scintigraphy | No |
| Ambulatory blood pressure monitoring, Electrocardiogram | Electrocardiogram, Ambulatory blood pressure monitoring | Yes |
| Brain CT scan, Electrocardiogram | Electrocardiogram | In part |
| Electrocardiogram, Echocardiogram, Exercise stress test, Myocardial perfusion scintigraphy, Coronary angiography, Chest X-ray | Echocardiogram, Ambulatory blood pressure monitoring, Cardiac MRI | In part |
| Head CT, Head MRI | Ambulatory blood pressure monitoring | No |
| Electrocardiogram, Myocardial perfusion scintigraphy, Coronary angiography | Exercise stress test, 24-hour Holter monitoring, Esophagogastroduodenoscopy | In part |
| Echocardiogram, Myocardial perfusion scintigraphy, Electrocardiogram | Two-dimensional echocardiogram | In part |
| Coronary CT, Echocardiogram, Chest X-ray, Electrocardiogram | Two-dimensional echocardiogram | In part |
| Echocardiogram, Chest X-ray, Electrocardiogram | Two-dimensional echocardiogram, Exercise stress test | In part |
| Brain CT scan, Electrocardiogram | Ambulatory blood pressure monitoring, Exercise stress test, Transthoracic echocardiogram with Doppler | No |
| Electrocardiogram, Echocardiogram, Exercise stress test, Myocardial perfusion scintigraphy, Coronary angiography | Exercise stress test | In part |
| Electrocardiogram, Echocardiogram, Exercise stress test, Myocardial perfusion scintigraphy, Coronary angiography | Echocardiogram with Doppler, Myocardial perfusion scintigraphy | In part |
| Venous Doppler ultrasound of the lower extremities | Duplex venous scan of the lower extremities | Yes |
| Electrocardiogram, Echocardiography, Chest X-ray, Exercise stress test, 24-hour Holter monitoring | Transthoracic echocardiogram, Myocardial perfusion scintigraphy | In part |
| Electrocardiogram, Echocardiogram, Exercise stress test, Myocardial perfusion scintigraphy, Coronary angiography, Chest X-ray | Exercise stress test, Transthoracic echocardiogram | In part |
| Electroencephalogram, Audiometry, Electrocardiogram | Ambulatory blood pressure monitoring, Echocardiogram | No |
| Echocardiogram, Chest X-ray, Exercise stress test | Echocardiogram, Chest X-ray | In part |
| Electrocardiogram, Coronary angiography, Vascular ultrasound, Exercise stress test | Echocardiogram | No |
| Electrocardiogram, Echocardiogram, Exercise stress test | Electrocardiogram, Echocardiogram, Exercise stress test | Yes |
| Electrocardiogram, Echocardiogram, Chest X-ray | Echocardiogram, Chest X-ray | In part |
| Electrocardiogram, Coronary angiography, Echocardiogram, Exercise stress test | Exercise stress test, Transthoracic echocardiogram, Myocardial perfusion scintigraphy | In part |
| Echocardiogram, 24-hour Holter monitoring, Exercise stress test | Echocardiogram, 24-hour Holter monitoring | In part |
| Echocardiogram, Exercise stress test, Myocardial perfusion scintigraphy | Echocardiogram, Myocardial perfusion scintigraphy | In part |
| Electrocardiogram, Echocardiogram, Chest X-ray, Esophagogastroduodenoscopy | Electrocardiogram, Transthoracic echocardiogram | In part |
| Electrocardiogram, Echocardiogram, Exercise stress test, Chest X-ray | Transthoracic echocardiogram, Electrocardiogram | In part |
| Electrocardiogram, Echocardiogram, Chest X-ray, Esophagogastroduodenoscopy | Exercise stress test, Esophagogastroduodenoscopy | In part |
| Electrocardiogram, Echocardiogram, Chest X-ray | Electrocardiogram, Myocardial perfusion scintigraphy | In part |
| Electrocardiogram, Echocardiogram, Exercise stress test, Coronary angiography | Electrocardiogram, Chest X-ray | In part |
| Electrocardiogram, Echocardiogram, Exercise stress test, Coronary angiography | - | No |
| Echocardiogram, Electrocardiogram, Chest X-ray, Venous Doppler ultrasound | Echocardiogram | In part |
| Echocardiogram, Electrocardiogram, Chest X-ray, Venous Doppler ultrasound | Echocardiogram | In part |
| Electrocardiogram, Myocardial perfusion scintigraphy, Exercise stress test, Echocardiogram, Chest X-ray, Coronary angiography | Electrocardiogram, Myocardial perfusion scintigraphy | In part |
| Electrocardiogram, Chest X-ray, Echocardiogram, Myocardial perfusion scintigraphy | Transthoracic echocardiogram, Myocardial perfusion scintigraphy | In part |
| Electrocardiogram, Echocardiogram, Chest X-ray, Ambulatory blood pressure monitoring | Electrocardiogram | In part |
| Electrocardiogram, Chest X-ray, Echocardiogram | Electrocardiogram, Transthoracic echocardiogram | In part |
| Electrocardiogram, Chest X-ray, Echocardiogram, Chest CT, Cardiac MRI | Myocardial perfusion scintigraphy | No |
| Electrocardiogram, Exercise stress test, Echocardiogram, Coronary angiography | Myocardial perfusion scintigraphy | In part |
| Electrocardiogram, Echocardiogram, Exercise stress test, Coronary angiography | 24-hour Holter monitoring | No |
| Electrocardiogram | Echocardiogram | No |
| Echocardiogram, Electrocardiogram, Venous Doppler ultrasound, Chest X-ray | - | No |
| Chest X-ray, Echocardiogram, Chest CT | - | No |
| Electrocardiogram, 24-hour Holter monitoring, Electroencephalogram | 24-hour Holter monitoring, Echocardiogram | In part |
| Doppler echocardiography, Electrocardiogram, Exercise stress test, Chest X-ray | Electrocardiogram | In part |
| Coronary angiography, Echocardiogram, Coronary CT, Electrocardiogram | Echocardiogram | In part |
| Coronary angiography, CT angiography, Cardiac magnetic resonance imaging, Electrocardiogram, Exercise stress test, Echocardiogram | Electrocardiogram, Echocardiogram | In part |
| Coronary angiography, CT angiography, Cardiac MRI, Echocardiogram, Exercise stress test, 24-hour Holter monitoring | Electrocardiogram, Echocardiogram with Doppler | In part |
| Electrocardiogram, Echocardiogram, Chest X-ray, 24-hour Holter monitoring, Cardiac magnetic resonance imaging, Cardiac catheterization | Echocardiogram, Myocardial perfusion scintigraphy | In part |
| Electrocardiogram, Echocardiogram, Chest X-ray, 24-hour Holter monitoring, Myocardial perfusion scintigraphy | Electrocardiogram, Transthoracic echocardiogram | In part |
| Electrocardiogram, Exercise stress test, Echocardiogram, Myocardial perfusion scintigraphy, Coronary angiography | Exercise stress test, Chest X-ray | In part |
| Electrocardiogram, Exercise stress test, 24-hour Holter monitoring, Cardiac magnetic resonance imaging | 24-hour Holter monitoring, Myocardial perfusion scintigraphy, Transthoracic echocardiogram with Doppler, Exercise stress test | In part |
| Head MRI, Electrocardiogram, Myocardial perfusion scintigraphy | Polysomnography, Aorto-renal Doppler ultrasound | No |
| Echocardiogram, Ventilation–perfusion lung scan, Chest CT, Cardiac magnetic resonance imaging, Polysomnography | Exercise stress test, Chest X-ray, Echocardiogram with Doppler | No |
| Myocardial perfusion scintigraphy, Echocardiogram, CT angiography, Cardiac CT, Cardiac catheterization, 24-hour Holter monitoring | Exercise stress test, 24-hour Holter monitoring, Echocardiogram with Doppler | In part |
| Electrocardiogram, Echocardiogram, Chest X-ray, 24-hour Holter monitoring, Cardiac magnetic resonance imaging, Cardiac catheterization | Echocardiogram, 24-hour Holter monitoring | In part |
| Echocardiogram with Doppler | Echocardiogram with Doppler | Yes |
| Electrocardiogram, Echocardiogram, Chest X-ray | Myocardial perfusion scintigraphy | No |
| Electrocardiogram, Echocardiogram, Exercise stress test, 24-hour Holter monitoring | Exercise stress test, Echocardiogram | In part |
| Electrocardiogram, Exercise stress test, Echocardiogram, Coronary angiography | Echocardiogram | In part |
| Brain CT scan, Ophthalmic examination, Ambulatory blood pressure monitoring | Abdominal ultrasonography, Echocardiogram | No |
| Electrocardiogram, Echocardiogram, Exercise stress test, Chest X-ray | Exercise stress test | In part |
| Electrocardiogram, Echocardiogram, Exercise stress test, Chest X-ray, Myocardial perfusion scintigraphy | Myocardial perfusion scintigraphy | In part |
| Electrocardiogram, Echocardiogram, Exercise stress test, Chest X-ray, Myocardial perfusion scintigraphy | Electrocardiogram, Myocardial perfusion scintigraphy | In part |
| Electrocardiogram, Echocardiogram, Chest X-ray, 24-hour Holter monitoring, Myocardial perfusion scintigraphy | Echocardiogram, 24-hour Holter monitoring, Polysomnography | In part |
| Electrocardiogram, Echocardiogram, Chest X-ray, 24-hour Holter monitoring, Myocardial perfusion scintigraphy | Echocardiogram, Abdominal ultrasonography | In part |
| Electrocardiogram, Echocardiogram, Chest X-ray, 24-hour Holter monitoring | Chest X-ray, Electrocardiogram, 24-hour Holter monitoring, Echocardiogram | In part |
| Chest X-ray, Electrocardiogram, Echocardiogram, Spirometry, Exercise stress test | Electrocardiogram, Echocardiogram | In part |
| Electrocardiogram, Echocardiogram, Chest X-ray, 24-hour Holter monitoring, Myocardial perfusion scintigraphy | Echocardiogram | In part |
| Electrocardiogram, Echocardiogram, Exercise stress test, Chest X-ray, Myocardial perfusion scintigraphy | Chest X-ray, Electrocardiogram, Echocardiogram with Doppler | In part |
| Electrocardiogram, Transthoracic echocardiography, Chest X-ray, 24-hour Holter monitoring, Myocardial perfusion scintigraphy | Electrocardiogram, Chest X-ray, Echocardiogram | In part |
| Ambulatory blood pressure monitoring, Electrocardiogram | Electrocardiogram | In part |
| Electrocardiogram, Echocardiogram, Exercise stress test | Electrocardiogram, Echocardiogram | In part |
| Electrocardiogram, Ambulatory blood pressure monitoring | Echocardiogram, Chest X-ray | No |
| Electrocardiogram, Echocardiogram, Exercise stress test, Chest X-ray, Spirometry, Myocardial perfusion scintigraphy | Electrocardiogram, Transthoracic echocardiogram | In part |
| Electrocardiogram, Exercise stress test, Chest X-ray, Myocardial perfusion scintigraphy | Echocardiogram with Doppler | No |
| Electrocardiogram, Echocardiogram, Exercise stress test, Chest X-ray, Myocardial perfusion scintigraphy | Electrocardiogram, Echocardiogram | In part |
| Electrocardiogram, Echocardiogram, Myocardial perfusion scintigraphy, Spirometry, Chest X-ray | Electrocardiogram, Echocardiogram, 24-hour Holter monitoring | In part |
| Electrocardiogram, Echocardiogram, Exercise stress test, Chest X-ray, Myocardial perfusion scintigraphy | Echocardiogram | In part |
| Electrocardiogram, Echocardiogram, Myocardial perfusion scintigraphy, Coronary angiography, Coronary CT, Cardiac magnetic resonance imaging | Echocardiogram | In part |
| Chest X-ray, Electrocardiogram, Echocardiogram, Exercise stress test, Cardiac magnetic resonance imaging, Myocardial perfusion scintigraphy, Cardiac catheterization | Electrocardiogram, Echocardiogram, Polysomnography | In part |
| Electrocardiogram, Echocardiogram, Exercise stress test, 24-hour Holter monitoring, Myocardial perfusion scintigraphy, Coronary angiography | Electrocardiogram, Exercise stress test, Echocardiogram with Doppler, Myocardial perfusion scintigraphy | In part |
| Electrocardiogram, Echocardiogram, Chest X-ray, Cardiac magnetic resonance imaging, Myocardial perfusion scintigraphy, Cardiac catheterization | Chest X-ray, Abdominal ultrasound, Echocardiogram | In part |
| Electrocardiogram, Echocardiogram, Exercise stress test, Cardiac magnetic resonance imaging, Abdominal CT | Echocardiogram | In part |
| Echocardiogram, 24-hour Holter monitoring, Exercise stress test, Cardiac magnetic resonance imaging, Chest CT, Spirometry | Echocardiogram, 24-hour Holter monitoring, Chest X-ray | In part |
| Electrocardiogram, Echocardiogram, Myocardial perfusion scintigraphy, Coronary angiography, Cardiac magnetic resonance imaging, Coronary CT | Electrocardiogram, Myocardial perfusion scintigraphy | In part |
| Echocardiogram, Electrocardiogram, Chest X-ray, Exercise stress test, Cardiac magnetic resonance imaging | Echocardiogram with Doppler, Chest X-ray | In part |
| Echocardiogram, Electrocardiogram, Chest X-ray, Exercise stress test | Ambulatory blood pressure monitoring, Echocardiogram with Doppler, Chest X-ray | In part |
| Echocardiogram, Electrocardiogram, Chest X-ray, Exercise stress test | Exercise stress test, Echocardiogram, Electrocardiogram | In part |
| Electrocardiogram, Echocardiogram, Chest X-ray, Exercise stress test, Cardiac MRI, Cardiac catheterization | Echocardiogram with Doppler, Chest X-ray | In part |
| Electrocardiogram, Echocardiogram, Myocardial perfusion scintigraphy, Coronary angiography, Cardiac magnetic resonance imaging | Myocardial perfusion scintigraphy, Echocardiogram with Doppler, Exercise stress test, 24-hour Holter monitoring | In part |
