# Supplementary material for: ChatGPT in Public Cardiovascular Healthcare: Accuracy, Limitations, and Implications
Source: J Eval Clin Pract. 2026 May 3;32:e70465. doi: 10.1111/jep.70465 (PMC13135820; doi:10.1111/jep.70465)
Supplement: Supplementary file 3 — Supporting Table 3 [file JEP-32-0-s007.docx]

**Supplementary Table 3:** Questions designed for ChatGPT were formulated following this model: “For a patient [Man (M) or Woman (W)], aged (xx) with (Risk factors) and presenting (Chief complaint)-as seen in Supplementary Table 1, what laboratory tests should I request?”

| **Reply from ChatGPT** | **Recorded in the medical file** | **Was ChatGPT successful?** |
| --- | --- | --- |
| Lipid profile, Hemogram, Fasting blood glucose, Hemoglobin A1c, Creatinine and glomerular filtration rate, Uric acid, Electrolytes, C-reactive protein, D-dimer, Liver function tests | Hemogram, Sodium, Potassium, Magnesium, Creatinine, Urea, Total cholesterol and fractions, Triglycerides, Fasting glucose, Hemoglobin A1c, Vitamin B12, Vitamin D, Aspartate Aminotransferase, Alanine Aminotransferase, Alkaline phosphatase, Gamma-glutamyl transferase, Thyroid Stimulating Hormone | In part |
| Lipid profile, Hemogram, Fasting blood glucose, Hemoglobin A1c, Creatinine and glomerular filtration rate, Uric acid, Electrolytes, C-reactive protein, D-dimer, Liver function tests, Blood ethanol level, Thyroid-stimulating hormone, Free thyroxine | Creatinine, Urea | In part |
| Troponin, CK-MB, Lipid profile, Fasting blood glucose, Hemogram, Electrolytes | Hemogram, Total cholesterol and fractions, Triglycerides, Fasting glucose, Hemoglobin A1c | In part |
| Hemogram, Fasting blood glucose, Hemoglobin A1c, Renal function, Electrolytes, Troponin, CK-MB, Prothrombin time, Activated partial thromboplastin time, C-reactive protein, Thyroid-stimulating hormone | Hemogram, Sodium, Potassium, Creatinine, Urea, Total cholesterol and fractions, Aspartate Aminotransferase, Alanine Aminotransferase, Triglycerides, Fasting glucose, Hemoglobin A1c, Thyroid Stimulating Hormone, Free Thyroxine, Vitamin B12 | In part |
| Hemogram, Lipid profile, Fasting blood glucose, Hemoglobin A1c, Urea and creatinine, Electrolytes, B-type natriuretic peptide, Coagulation profile, Liver function tests, Urinalysis, Arterial blood gas analysis | - | No |
| Lipid profile, Hemogram, Fasting blood glucose, Hemoglobin A1c, Renal function, Electrolytes, Liver function tests, Thyroid-stimulating hormone, Free thyroxine | - | No |
| Hemogram, Fasting blood glucose, Hemoglobin A1c, Renal function, Liver function tests, Electrolytes, C-reactive protein, D-dimer, Lipid profile, Thyroid-stimulating hormone | Thyroid Stimulating Hormone, Free Thyroxine, Total cholesterol and fractions, Fasting glucose, Hemoglobin A1c | In part |
| Lipid profile, Fasting blood glucose, CK-MB, Troponin, C-reactive protein, Renal function, Liver function tests, Electrolytes, Coagulation profile | Hemogram, Sodium, Potassium, Magnesium, Creatinine, Urea, Total cholesterol and fractions, Triglycerides, Fasting glucose, Hemoglobin A1c, Aspartate Aminotransferase, Alanine Aminotransferase, Alkaline phosphatase, Gamma-glutamyl transferase, Free thyroxine, Thyroid Stimulating Hormone | In part |
| Lipid profile, Cardiac biomarkers | - | No |
| Lipid profile, Fasting blood glucose, High-sensitivity C-reactive protein, Renal function tests, Electrolytes, Hemogram, Coagulation profile, Cardiac biomarkers | - | No |
| Troponin, CK-MB, Liver enzymes, Creatinine and renal function, Hemogram, Electrolytes, Glucose, Lipid profile | - | No |
| Hemogram, Blood glucose, Hemoglobin A1c, Lipid profile, Liver function tests, Electrolytes, Renal function, C-reactive protein | - | No |
| Lipid profile, Hemogram, Fasting blood glucose, Renal function, Liver function tests, C-reactive protein, Electrolytes, Prothrombin time, Activated partial thromboplastin time, Thyroid-stimulating hormone, Free thyroxine, Urinalysis | - | No |
| Hemogram, Fasting blood glucose, Lipid profile, Renal function, Electrolytes, Thyroid-stimulating hormone, Free thyroxine | - | No |
| Hemogram, Troponin, Lipid profile, Electrolytes, Renal function, Fasting blood glucose, Hemoglobin A1c, Liver function tests | Hemogram, Sodium, Potassium, Magnesium, Creatinine, Urea, Total Cholesterol and Fractions, Triglycerides, Fasting Blood Glucose, Hemoglobin A1c | In part |
| Hemogram, Renal and liver function tests, Lipid profile, Fasting blood glucose, Thyroid-stimulating hormone, Free thyroxine | Hemogram, Sodium, Potassium, Magnesium, Creatinine, Urea, Total Cholesterol and Fractions, Triglycerides, Fasting Blood Glucose, Hemoglobin A1c, Aspartate Aminotransferase, Alanine Aminotransferase, Alkaline Phosphatase, Gamma-Glutamyl Transferase | In part |
| Hemogram, Troponin | Hemograma, Sódio, Potássio, Creatinina, Ureia, Colesterol total e frações, TGO, TGP, Triglicerides, Glicemia jejum, HbA1c | In part |
| Hemogram, Troponin, Renal function | NT to BNT, Creatinine, Potassium | In part |
| Activated partial thromboplastin time, Prothrombin time | Hemogram, Fasting Blood Glucose, Hemoglobin A1c, Total Cholesterol and Fractions, Creatinine, Urea, Aspartate Aminotransferase, Alanine Aminotransferase | No |
| Cardiac biomarkers, Renal function, Liver function tests, Hemogram, Electrolytes | Hemogram, Fasting Blood Glucose, Hemoglobin A1c, Lipid Profile, Thyroid Stimulating Hormone, Free T4, Urea, Creatinine | In part |
| Coagulation profile, Troponin, Fasting blood glucose | Hemogram, Sodium, Potassium, Creatinine, Urea, Total Cholesterol and Fractions, Aspartate Aminotransferase, Alanine Aminotransferase, Triglycerides, Fasting Blood Glucose, Hemoglobin A1c | No |
| Lipid profile, Troponin, CK-MB | Hemogram, Sodium, Potassium, Magnesium, Creatinine, Urea, Total Cholesterol and Fractions, Triglycerides, Fasting Blood Glucose, Hemoglobin A1c | In part |
| Hemogram, Coagulation profile, Troponin, Fasting blood glucose, Renal function, C-reactive protein, Liver function tests | Fasting Blood Glucose, Hemoglobin A1c, Total Cholesterol and Fractions, Triglycerides, Thyroid Stimulating Hormone, Urea, Creatinine | In part |
| Hemogram, D-dimer, Fasting blood glucose, Coagulation profile | - | No |
| Hemogram, Fasting blood glucose, Lipid profile, B-type natriuretic peptide, Renal function, Liver function tests, C-reactive protein | Hemogram, Sodium, Potassium, Magnesium, Creatinine, Urea, Total Cholesterol and Fractions, Triglycerides, Fasting Blood Glucose, Aspartate Aminotransferase, Alanine Aminotransferase, Alkaline Phosphatase, Gamma-Glutamyl Transferase | In part |
| Troponin, Hemogram, Renal function, Liver function tests, Lipid profile, C-reactive protein | Hemogram, Sodium, Potassium, Magnesium, Creatinine, Urea, Total Cholesterol and Fractions, Triglycerides | In part |
| Hemogram, Thyroid-stimulating hormone, Free thyroxine, Liver function tests, Lipid profile | Hemogram, Triglycerides, Total Cholesterol and Fractions, Fasting Blood Glucose, Hemoglobin A1c, Urea, Creatinine | In part |
| Hemogram, Fasting blood glucose, Liver function tests, Renal function, Lipid profile, Coagulation profile | Triglycerides, Total Cholesterol and Fractions, Fasting Blood Glucose, Hemoglobin A1c, Urea, Creatinine | In part |
| Hemogram, Lipid profile, Fasting blood glucose, Liver function tests, Renal function, Coagulation profile | Hemogram, Sodium, Potassium, Magnesium, Creatinine, Urea, Total Cholesterol and Fractions, Triglycerides, Hemoglobin A1c, Aspartate Aminotransferase, Alanine Aminotransferase, Alkaline Phosphatase, Gamma-Glutamyl Transferase, Uric Acid | In part |
| Hemogram, Electrolytes, Renal function, Lipid profile, Fasting blood glucose, C-reactive protein | - | No |
| Hemogram, Electrolytes, Renal function, Lipid profile, Fasting blood glucose, Hemoglobin A1c, Liver function tests, Coagulation profile | Hemogram, Electrolytes, Renal function, Lipid profile, Fasting blood glucose, Hemoglobin A1c, Liver function tests, Coagulation profile | Yes |
| Hemogram, Fasting blood glucose, Electrolytes, Renal function, Liver function tests, C-reactive protein, Troponin | Hemogram, Sodium, Potassium, Magnesium, Creatinine, Urea, Total Cholesterol and Fractions, Triglycerides, Fasting Blood Glucose, Hemoglobin A1c, Mean Corpuscular Hemoglobin Concentration, Mean Corpuscular Volume, Mean Corpuscular Hemoglobin, Red Cell Distribution Width, Hematocrit, Uric Acid, Aspartate Aminotransferase, Alanine Aminotransferase | In part |
| Hemogram, Fasting blood glucose, Hemoglobin A1c, Electrolytes, Renal function, Liver function tests | Hemogram, Fasting blood glucose, Hemoglobin A1c, Electrolytes, Renal function, Liver function tests | Yes |
| Hemogram, Fasting blood glucose, Electrolytes, Renal function, Liver function tests, C-reactive protein, Troponin, Arterial blood gas analysis, Coagulation profile | Hemogram, Sodium, Potassium, Magnesium, Creatinine, Urea, Total Cholesterol and Fractions, Triglycerides, Hemoglobin A1c, Aspartate Aminotransferase, Alanine Aminotransferase, Alkaline Phosphatase, Gamma-Glutamyl Transferase, Uric Acid | In part |
| Hemogram, Fasting blood glucose, Electrolytes, Renal function, Liver function tests, C-reactive protein, Troponin, CK-MB, Thyroid-stimulating hormone, Free thyroxine | Hemogram, Sodium, Potassium, Creatinine, Urea, Total Cholesterol and Fractions, Triglycerides, Fasting Blood Glucose, Hemoglobin A1c | In part |
| Lipid profile, Hemogram, Fasting blood glucose, Hemoglobin A1c, Creatinine and glomerular filtration rate, Uric acid, Electrolytes, C-reactive protein, D-dimer, Liver function tests | Hemogram, Sodium, Potassium, Magnesium, Creatinine, Urea, Total Cholesterol and Fractions, Triglycerides, Fasting Blood Glucose, Hemoglobin A1c | In part |
| Lipid profile, Hemogram, Fasting blood glucose, Hemoglobin A1c, Creatinine and glomerular filtration rate, Uric acid, Electrolytes, C-reactive protein, D-dimer, Liver function tests, Blood ethanol level, Thyroid-stimulating hormone, Free thyroxine | Fasting Blood Glucose, Hemoglobin A1c, Total Cholesterol and Fractions, Triglycerides, Hemogram, Prothrombin Time, Creatinine, Urea, Sodium, Potassium, Routine Urinalysis | In part |
| Lipid profile, Fasting blood glucose, Hemogram, Electrolytes | Total Cholesterol and Fractions, Fasting Blood Glucose, Hemoglobin A1c, Triglycerides, Creatinine, Urea | Yes |
| Electrolytes, Troponin, CK-MB, Prothrombin time, Activated partial thromboplastin time, C-reactive protein, Thyroid-stimulating hormone | Hemogram, Urea, Creatinine, Sodium, Potassium, Total Cholesterol, Triglycerides, Thyroid Stimulating Hormone, Fasting Blood Glucose, B-type Natriuretic Peptide | No |
| Hemogram, Lipid profile, Fasting blood glucose, Hemoglobin A1c, Urea and creatinine, Electrolytes, B-type natriuretic peptide, Coagulation profile, Liver function tests, Urinalysis, Arterial blood gas analysis | - | No |
| Lipid profile, Hemogram, Fasting blood glucose, Hemoglobin A1c, Renal function, Electrolytes, Liver function tests, Thyroid-stimulating hormone, Free thyroxine | Creatinine, Potassium, Total Cholesterol and Fractions, Triglycerides, Aspartate Aminotransferase, Alanine Aminotransferase, Fasting Blood Glucose, Hemoglobin A1c | In part |
| Hemogram, Fasting blood glucose, Hemoglobin A1c, Renal function, Liver function tests, Electrolytes, C-reactive protein, D-dimer, Lipid profile, Thyroid-stimulating hormone | - | No |
| Lipid profile, Fasting blood glucose, CK-MB, Troponin, C-reactive protein, Renal function, Liver function tests, Electrolytes, Coagulation profile | Urea, Creatinine, Potassium, Sodium, Fasting Blood Glucose, Hemoglobin A1c, Triglycerides, Total Cholesterol and Fractions | Yes |
| Lipid profile, Cardiac biomarkers | - | No |
| Lipid profile, Fasting blood glucose, High-sensitivity C-reactive protein, Renal function tests, Electrolytes, Hemogram, Coagulation profile, Cardiac biomarkers | Proteinuria, Creatinine | In part |
| Troponin, CK-MB, Liver enzymes, Creatinine and renal function, Hemogram, Electrolytes, Glucose, Lipid profile | Lipid Profile, Glucose, Electrolytes, Hemogram, Creatinine and Renal Function, Liver Enzymes, CK-MB, Troponin | Yes |
| Hemogram, Blood glucose, Hemoglobin A1c, Lipid profile, Liver function tests, Electrolytes, Renal function, C-reactive protein | Hemograma, Sódio, Potássio, Mganésio, Creatinina, Ureia, Colesterol total e frações, Triglicerides, Glicemia jejum, Hemoglobina glicada, Vit B12, Vit D, TGO, TGP, FA, GGT, Uric Acid | In part |
| Lipid profile, Hemogram, Fasting blood glucose, Renal function, Liver function tests, C-reactive protein, Electrolytes, Prothrombin time, Activated partial thromboplastin time, Thyroid-stimulating hormone, Free thyroxine, Urinalysis | Hemogram, Fasting Blood Glucose, Hemoglobin A1c, Total Cholesterol and Fractions, Triglycerides, Total Creatine Kinase | In part |
| Hemogram, Fasting blood glucose, Lipid profile, Renal function, Electrolytes, Thyroid-stimulating hormone, Free thyroxine | - | No |
| Hemogram, Troponin, Lipid profile, Electrolytes, Renal function, Fasting blood glucose, Hemoglobin A1c, Liver function tests | - | No |
| Hemogram, Renal and liver function tests, Lipid profile, Fasting blood glucose, Thyroid-stimulating hormone, Free thyroxine | Hemogram, Total Cholesterol and Fractions, Triglycerides, Fasting Blood Glucose, Hemoglobin A1c, Creatinine, Urea, Sodium, Potassium, Phosphorus, Magnesium, Uric Acid, Calcium | In part |
| Hemogram, Troponin | - | No |
| Hemogram, Troponin, Renal function | - | No |
| Glucose, Hemoglobin A1c, Hemogram, Creatinine, Potassium, Uric Acid, Aspartate Aminotransferase, Alanine Aminotransferase, Total Cholesterol and Fractions | Hemogram, Creatinine, Potassium, Uric Acid, Glucose, Hemoglobin A1c, Aspartate Aminotransferase, Alanine Aminotransferase, Total Cholesterol and Fractions | Yes |
| Cardiac biomarkers, Renal function, Liver function tests, Hemogram, Electrolytes | - | No |
| Hemogram, Lipid profile, Renal function | Hemogram, Fasting Blood Glucose, Hemoglobin A1c, Total Cholesterol and Fractions, Triglycerides, Creatinine, Urea, Sodium, Potassium, Magnesium | In part |
| Lipid profile, Troponin, CK-MB | Hemogram, Sodium, Potassium, Magnesium, Creatinine, Urea, Total Cholesterol and Fractions, Triglycerides, Fasting Blood Glucose, Hemoglobin A1c, Aspartate Aminotransferase, Alanine Aminotransferase, Alkaline Phosphatase, Gamma-Glutamyl Transferase | In part |
| Hemogram, Coagulation profile, Troponin, Fasting blood glucose, Renal function, C-reactive protein, Liver function tests | Hemogram, Sodium, Potassium, Magnesium, Creatinine, Urea, Total Cholesterol and Fractions, Triglycerides, Fasting Blood Glucose, Hemoglobin A1c, Aspartate Aminotransferase, Alanine Aminotransferase, Alkaline Phosphatase, Gamma-Glutamyl Transferase | In part |
| Hemogram, D-dimer, Fasting blood glucose, Coagulation profile | Hemogram, Sodium, Potassium, Magnesium, Creatinine, Urea, Total Cholesterol and Fractions, Triglycerides, Fasting Blood Glucose, Hemoglobin A1c, Aspartate Aminotransferase, Alanine Aminotransferase, Alkaline Phosphatase | In part |
| Hemogram, Fasting blood glucose, Lipid profile, B-type natriuretic peptide, Renal function, Liver function tests, C-reactive protein | Hemogram, Sodium, Potassium, Magnesium, Creatinine, Urea, Total Cholesterol and Fractions, Triglycerides, Fasting Blood Glucose, Hemoglobin A1 | In part |
| Troponin, Hemogram, Renal function, Liver function tests, Lipid profile, C-reactive protein | Hemogram, Sodium, Potassium, Magnesium, Creatinine, Urea, Total Cholesterol and Fractions, Triglycerides, Fasting Blood Glucose, Hemoglobin A1c | In part |
| Hemogram, Thyroid-stimulating hormone, Free thyroxine, Liver function tests, Lipid profile | Hemogram, Sodium, Potassium, Magnesium, Creatinine, Urea | In part |
| Hemogram, Fasting blood glucose, Liver function tests, Renal function, Lipid profile, Coagulation profile | Hemogram, Sodium, Potassium, Magnesium, Creatinine, Urea, Total Cholesterol and Fractions, Triglycerides, Fasting Blood Glucose, Hemoglobin A1c, Aspartate Aminotransferase, Alanine Aminotransferase | In part |
| Hemogram, Lipid profile, Fasting blood glucose, Liver function tests, Renal function, Coagulation profile | - | No |
| Hemogram, Electrolytes, Renal function, Lipid profile, Fasting blood glucose, C-reactive protein | - | No |
| Hemogram, Electrolytes, Renal function, Lipid profile, Fasting blood glucose, Hemoglobin A1c, Liver function tests, Coagulation profile | Urea, Creatinine, Potassium | No |
| Hemogram, Fasting blood glucose, Electrolytes, Renal function, Liver function tests, C-reactive protein, Troponin | Total Cholesterol and Fractions, Creatinine, Fasting Blood Glucose, Potassium, Triglycerides, Ferritin, Vitamin B12, Total Creatine Kinase | In part |
| Hemogram, Fasting blood glucose, Hemoglobin A1c, Electrolytes, Renal function, Liver function tests | Hemogram, Sodium, Potassium, Magnesium, Creatinine, Urea, Total Cholesterol and Fractions, Triglycerides, Fasting Blood Glucose, Hemoglobin A1c | In part |
| Hemogram, Fasting blood glucose, Electrolytes, Renal function, Liver function tests, C-reactive protein, Troponin, Arterial blood gas analysis, Coagulation profile | Uric Acid, Fasting Blood Glucose, Hemoglobin A1c, Triglycerides, Total Cholesterol and Fractions | In part |
| Hemogram, Fasting blood glucose, Electrolytes, Renal function, Liver function tests, C-reactive protein, Troponin, CK-MB, Thyroid-stimulating hormone, Free thyroxine | Hemogram, Sodium, Potassium, Magnesium, Creatinine, Urea, Total Cholesterol and Fractions, Triglycerides, Fasting Blood Glucose, Hemoglobin A1c | In part |
| Lipid profile, Hemogram, Fasting blood glucose, Hemoglobin A1c, Creatinine and glomerular filtration rate, Uric acid, Electrolytes, C-reactive protein, D-dimer, Liver function tests | - | No |
| Lipid profile, Hemogram, Fasting blood glucose, Hemoglobin A1c, Creatinine and glomerular filtration rate, Uric acid, Electrolytes, C-reactive protein, D-dimer, Liver function tests, Blood ethanol level, Thyroid-stimulating hormone, Free thyroxine | Hemogram, Sodium, Potassium, Magnesium, Creatinine, Urea, Total Cholesterol and Fractions, Triglycerides, Hemoglobin A1c, Aspartate Aminotransferase, Alanine Aminotransferase | In part |
| Troponin, CK-MB, Lipid profile, Fasting blood glucose, Hemogram, Electrolytes | Hemogram, Sodium, Potassium, Magnesium, Creatinine, Urea, Total Cholesterol and Fractions, Triglycerides, Fasting Blood Glucose, Hemoglobin A1c | In part |
| Hemogram, Fasting blood glucose, Hemoglobin A1c, Renal function, Electrolytes, Troponin, CK-MB, Prothrombin time, Activated partial thromboplastin time, C-reactive protein, Thyroid-stimulating hormone | Creatinine, Urea, Hemoglobin A1c, Sodium, Potassium | In part |
| Hemogram, Lipid profile, Fasting blood glucose, Hemoglobin A1c, Urea and creatinine, Electrolytes, B-type natriuretic peptide, Coagulation profile, Liver function tests, Urinalysis, Arterial blood gas analysis | - | No |
| Lipid profile, Hemogram, Fasting blood glucose, Hemoglobin A1c, Renal function, Electrolytes, Liver function tests, Thyroid-stimulating hormone, Free thyroxine | Hemogram, Sodium, Potassium, Magnesium, Creatinine, Urea, Total Cholesterol and Fractions, Triglycerides, Fasting Blood Glucose, Hemoglobin A1c, Aspartate Aminotransferase, Alanine Aminotransferase, Alkaline Phosphatase, Gamma-Glutamyl Transferase | In part |
| Hemogram, Fasting blood glucose, Hemoglobin A1c, Renal function, Liver function tests, Electrolytes, C-reactive protein, D-dimer, Lipid profile, Thyroid-stimulating hormone | Hemogram, Triglycerides, Total Cholesterol and Fractions, Fasting Blood Glucose, Hemoglobin A1c | Yes |
| Lipid profile, Fasting blood glucose, CK-MB, Troponin, C-reactive protein, Renal function, Liver function tests, Electrolytes, Coagulation profile | Hemogram, Fasting Blood Glucose, Hemoglobin A1, Total Cholesterol and Fractions, Triglycerides, Urea, Creatinine, Sodium, Potassium, Routine Urinalysis | In part |
| Lipid profile, Cardiac biomarkers | Creatine Kinase, Aldolase, C-Reactive Protein, Triglycerides, Total Cholesterol and Fractions | In part |
| Lipid profile, Fasting blood glucose, High-sensitivity C-reactive protein, Renal function tests, Electrolytes, Hemogram, Coagulation profile, Cardiac biomarkers | Fasting Blood Glucose, Hemoglobin A1c | No |
| Troponin, CK-MB, Liver enzymes, Creatinine and renal function, Hemogram, Electrolytes, Glucose, Lipid profile | Hemogram, Sodium, Potassium, Magnesium, Creatinine, Urea, Total Cholesterol and Fractions, Triglycerides, Fasting Blood Glucose, Hemoglobin A1c | Yes |
| Hemogram, Blood glucose, Hemoglobin A1c, Lipid profile, Liver function tests, Electrolytes, Renal function, C-reactive protein | Hemogram, Triglycerides, Total Cholesterol and Fractions, Fasting Blood Glucose, Hemoglobin A1c | In part |
| Fasting Blood Glucose, Hemoglobin A1c, Total Cholesterol and Fractions, Triglycerides, Hemogram, Sodium, Potassium, Magnesium, Creatinine, Urea, Thyroid Stimulating Hormone, Free T4 | Hemogram, Sodium, Potassium, Magnesium, Creatinine, Urea, Total Cholesterol and Fractions, Triglycerides, Fasting Blood Glucose, Hemoglobin A1c, Thyroid Stimulating Hormone, Free T4 | Yes |
| Hemogram, Fasting blood glucose, Lipid profile, Renal function, Electrolytes, Thyroid-stimulating hormone, Free thyroxine | Hemogram, Urea, Creatinine, Hemoglobin A1c, Fasting Blood Glucose, Total Cholesterol and Fractions, Triglycerides, Potassium, B-type Natriuretic Peptide, Total and Free Prostate-Specific Antigen, Uric Acid | In part |
| Hemogram, Troponin, Lipid profile, Electrolytes, Renal function, Fasting blood glucose, Hemoglobin A1c, Liver function tests | Hemogram, Total Cholesterol and Fractions, Hemoglobin A1c, Fasting Blood Glucose, Triglycerides, Prostate-Specific Antigen | In part |
| Hemogram, Renal and liver function tests, Lipid profile, Fasting blood glucose, Thyroid-stimulating hormone, Free thyroxine | Hemogram, Fasting Blood Glucose, Hemoglobin A1c, Urea, Creatinine, Potassium, Uric Acid, Total Cholesterol and Fractions, Aspartate Aminotransferase, Alanine Aminotransferase | In part |
| Hemogram, Sodium, Potassium, Creatinine, Hemoglobin A1c, Total Cholesterol and Fractions, Triglycerides | Total Cholesterol and Fractions, Creatinine, Hemoglobin A1c, Potassium, Sodium, Triglycerides, Hemogram | Yes |
| Hemogram, Troponin, Renal function | Hemoglobin A1c, Fasting Blood Glucose, Complete Blood Count, Total Cholesterol and Fractions, Creatinine, Urea, Potassium | In part |
| Activated partial thromboplastin time, Prothrombin time | Hemogram | In part |
| Cardiac biomarkers, Renal function, Liver function tests, Hemogram, Electrolytes | Hemogram, Urea, Creatinine, Sodium, Potassium, Magnesium, Calcium, Parathyroid Hormone, Vitamin D, Vitamin B12, Total cholesterol and fractions, Triglycerides, C-reactive protein, Hemoglobin A1c, Uric acid, Coagulogram, Free Thyroxine, Urinalysis, Urine culture | In part |
| Hemogram, Lipid profile, Renal function | Hemogram, Hemoglobin A1c, Fasting glucose, Triglycerides, Total cholesterol and fractions, Creatinine, Urea, Uric acid, Sodium, Potassium, Thyroid Stimulating Hormone, Free Thyroxine, Gamma-glutamyl transferase | In part |
| Lipid profile, Troponin, CK-MB | Vitamin D, Vitamin B12, Thyroid Stimulating Hormone, Aspartate Aminotransferase, Alanine Aminotransferase, Coagulogram, Fecal occult blood test | In part |
| Hemogram, Coagulation profile, Troponin, Fasting blood glucose, Renal function, C-reactive protein, Liver function tests | Hemogram, Fasting glucose and Hemoglobin A1c, Lipid profile, Creatinine and Urea, Aspartate Aminotransferase, Alanine Aminotransferase | In part |
| Hemogram, D-dimer, Fasting blood glucose, Coagulation profile | Hemogram, Fasting glucose, Hemoglobin A1c, Total cholesterol and fractions, Triglycerides, Creatinine, Urea, Sodium, Potassium, Magnesium | In part |
| Hemogram, Fasting blood glucose, Lipid profile, B-type natriuretic peptide, Renal function, Liver function tests, C-reactive protein | Hemogram, Fasting glucose, Hemoglobin A1c, Total cholesterol and fractions, Triglycerides, Creatinine, Urea, Sodium, Potassium | In part |
| Troponin, Hemogram, Renal function, Liver function tests, Lipid profile, C-reactive protein | Lipid profile, Fasting glucose, Hemoglobin A1c, Renal function | In part |
| Hemogram, Thyroid-stimulating hormone, Free thyroxine, Liver function tests, Lipid profile | Hemogram, Hemoglobin A1c, Fasting glucose, Triglycerides, Total cholesterol and fractions, Creatinine, Urea, Uric acid, Sodium, Potassium, Thyroid Stimulating Hormone, Free Thyroxine, Aspartate Aminotransferase, Alanine Aminotransferase, Gamma-glutamyl transferase, Vitamin D | In part |
| Hemogram, Fasting blood glucose, Liver function tests, Renal function, Lipid profile, Coagulation profile | Hemogram, Lipid profile, Fasting glucose, Hemoglobin A1c, Renal function | In part |
| Hemogram, Lipid profile, Fasting blood glucose, Liver function tests, Renal function, Coagulation profile | Hemogram, Sodium, Potassium, Magnesium, Creatinine, Urea, Total cholesterol and fractions, Triglycerides, Fasting glucose, Hemoglobin A1c, Aspartate Aminotransferase, Alanine Aminotransferase, Alkaline phosphatase, Gamma-glutamyl transferase | In part |
| Hemogram, Electrolytes, Renal function, Lipid profile, Fasting blood glucose, C-reactive protein | Hemogram, Fasting glucose, Hemoglobin A1c, Lipid profile, Creatinine, Urea | In part |
