# Supplementary material for: ChatGPT in Public Cardiovascular Healthcare: Accuracy, Limitations, and Implications
Source: J Eval Clin Pract. 2026 May 3;32:e70465. doi: 10.1111/jep.70465 (PMC13135820; doi:10.1111/jep.70465)
Supplement: Supplementary file 5 — Supporting Table 5 [file JEP-32-0-s008.docx]

**Supplementary Table 5:** Questions designed for DeepSeek were formulated following this model: “For a patient [Man (M) or Woman (W)], aged (xx) with (Risk factors) and presenting (Chief complaint), what is the diagnostic hypothesis?”

| **Sex** | **Age** | **Risk factors** | **Chief complaint** | **Reply from DeepSeek** | **Recorded in the medical file** | **Was DeepSeek**  **successful?** |
| --- | --- | --- | --- | --- | --- | --- |
| M | 63 | Smoker, stroke, systemic arterial hypertension, dyslipidemia, prediabetes, sedentary | Chest pain with tightness, particularly on the left side, worsening with minimal exertion and occurring daily | Angina | Chronic Ischemic Heart Disease | No |
| M | 65 | Systemic arterial hypertension, dyslipidemia, ex-smoker, sedentary, ex-alcoholism | Chest pain accompanied by dyspnea during physical exertion | Angina | Aortic Dissection | No |
| W | 78 | Sedentary | Left-sided chest pain with a burning sensation that worsens with physical exercise | Angina | Angina | Yes |
| W | 59 | Diabetes mellitus 2, dyslipidemia, systemic arterial hypertension, sedentary | Burning chest pain radiating to the left arm, exacerbated by physical exercise | Angina | Angina | Yes |
| W | 72 | Systemic arterial hypertension, diabetes mellitus 2, stroke | Bradycardia with the presence of edema in the lower extremities and dyspnea on moderate exertion | Heart Failure | Congestive Heart Failure | In part |
| W | 79 | Systemic arterial hypertension, diabetes mellitus 2, hypercholesterolemia | Orthostatic reflux associated with chest pain | Gastroesophageal Reflux Disease | Congestive Heart Failure | No |
| M | 75 | Systemic arterial hypertension, diabetes mellitus 2, ex-smoker, ex-alcoholism | Sensation of weakness associated with dyspnea | Heart Failure | Hypertensive Cardiomyopathy | No |
| W | 67 | Systemic arterial hypertension, dyslipidemia, ex-smoker | Intermittent chest pain radiating to the left arm unrelated to physical stress | Angina | Angina | Yes |
| M | 53 | Smoker, sedentary | Pain in the retrosternal region associated with fatigue and tachycardia on moderate exertion | Angina | Coronary Artery Disease | No |
| M | 59 | Systemic arterial hypertension, diabetes mellitus | Fatigue, precordial pain, and dyspnea with minimal exertion | Heart Failure | Chronic Ischemic Heart Disease | No |
| M | 73 | Diabetes mellitus, dyslipidemia, smoker | Burning chest pain on the left side associated with spikes in blood pressure | Angina | Systemic Arterial Hypertension | No |
| W | 55 | Systemic arterial hypertension, diabetes mellitus, dyslipidemia, hepatic steatosis | Dyspnea, constant chest pain, dry cough at night, fatigue with minimal exertion and dizziness | Heart Failure | Heart Failure with Preserved Ejection Fraction | In part |
| M | 61 | Stroke, dyslipidemia, ex-smoker, sedentary | Headache and nausea associated with elevated blood pressure | Hypertensive emergency | Systemic Arterial Hypertension | In part |
| W | 67 | Sedentary | Headache, vertigo, and edema in lower limbs | Chronic Venous Insufficiency | Systemic Arterial Hypertension | No |
| W | 39 | Systemic arterial hypertension | Stabbing chest pain with radiation to the left upper limb, associated with cough and dyspnea | Pulmonary Embolism | Heart Failure with Preserved Ejection Fraction | No |
| W | 66 | Dyslipidemia | Pain in the top of the head associated with edema in the lower limbs | Hypertensive crisis | Systemic Arterial Hypertension | In part |
| M | 60 | Ex-smoker, diabetes mellitus, systemic arterial hypertension | Chest pain and heartburn during physical activity | Angina | Gastroesophageal Reflux Disease | No |
| W | 79 | Dyslipidemia, systemic arterial hypertension, kidney failure, hypothyroidism | Dyspnea on moderate exertion associated with momentary precordial pain | Angina | Hypertensive Cardiomyopathy | No |
| W | 59 | Ex-smoker, acute myocardial infarction, dyslipidemia, systemic arterial hypertension | Epigastric pain, nausea, dizziness, palpitations associated with elevated blood pressure readings | Acute Myocardial Infarction | Systemic Arterial Hypertension | No |
| M | 76 | Sedentary, diabetes mellitus, dyslipidemia, systemic arterial hypertension | Dyspnea on significant exertion with orthopnea and edema in the lower extremities | Heart Failure | Left Bundle Branch Block | No |
| M | 64 | Stroke, ex-smoker, ex-alcoholism, dyslipidemia | Fall from one's own height without signs of syncope | Syncope | Systemic Arterial Hypertension | No |
| W | 40 | Sedentary | Dyspnea associated with sweating, tachycardia, and stabbing chest pain during moments of stress, unrelated to physical exertion | Panic disorder | Angina | No |
| W | 54 | Dyslipidemia, systemic arterial hypertension, sedentary | Chest pain with tightness and dyspnea related to physical exertion | Chronic Coronary Syndrome | Angina | No |
| W | 64 | Diabetes mellitus, dyslipidemia, systemic arterial hypertension | Pain in the left lower extremity accompanied by swelling and redness in the calf region | Deep Vein Thrombosis | Deep Vein Thrombosis | Yes |
| W | 60 | Systemic arterial hypertension, dyslipidemia, pre-diabetes | Chest pain with tightness and dyspnea on exertion associated with lower extremity edema and orthopnea | Heart Failure with Preserved Ejection Fraction | Congestive Heart Failure | In part |
| M | 59 | Acute myocardial infarction SST (2023), diabetes mellitus, systemic arterial hypertension, ex-smoker (5 pack-years), dyslipidemia | Chest pain and palpitations on moderate to heavy exertion | Angina | Angina | Yes |
| M | 70 | Dyslipidemia, sedentary | Episodes of vertigo preceded by chills and tremors associated with excessive fatigue | Angina | Systemic Arterial Hypertension | No |
| W | 76 | Systemic arterial hypertension, atrial fibrillation, dyslipidemia | Dyspnea on moderate exertion without precordial pain | Heart Failure | Congestive Heart Failure | In part |
| M | 64 | Diabetes mellitus, systemic arterial hypertension, dyslipidemia, acute myocardial infarction (2019), ex-smoker (11 pack-years), ex-alcoholism | Darkening of vision, pain in the chest, tingling in extremities, and a sensation of tightness and burning in the precordial region | Angina | Heart Failure with Preserved Ejection Fraction | No |
| W | 63 | Systemic arterial hypertension | Palpitations during moderate exertion and at night lasting for 30 minutes, denies dyspnea and precordial pain | Paroxysmal Supraventricular Tachycardia | Cardiac Arrhythmia | In part |
| M | 91 | Ex-smoker, alcoholism, systemic arterial hypertension, stroke | Dyspnea with minimal exertion, edema in lower extremities, and daily fatigue. Denies orthopnea, angina, or palpitations | Heart Failure | Congestive Heart Failure | In part |
| W | 44 | Systemic arterial hypertension, diabetes mellitus 2 | Chest pain with tightness on exertion, radiating to the left upper limb, associated with dyspnea, nausea, and palpitations | Angina | Angina | Yes |
| W | 67 | Systemic arterial hypertension, diabetes mellitus 2, smoking, alcoholism | Bradycardia and dyspnea on significant exertion | Chronic Chagasic Cardiomyopathy | Right Bundle Branch Block | No |
| W | 76 | Systemic arterial hypertension, stroke | Chest pain with tightness on moderate exertion that improves with rest | Angina | Angina | Yes |
| W | 58 | Systemic arterial hypertension | Burning chest pain with radiation to the cervical region, of mild intensity | Acute Coronary Syndrome | Systemic Arterial Hypertension | No |
| W | 66 | Diabetes mellitus 2, systemic arterial hypertension | Dyspnea and fatigue with minor exertion accompanied by edema in the lower extremities | Heart Failure | Congestive Heart Failure | In part |
| M | 80 | Systemic arterial hypertension, atrial fibrillation, coronary artery disease, heart failure | Dyspnea and fatigue on moderate exertion with difficulty swallowing | Heart Failure exacerbation | Heart Failure with Preserved Ejection Fraction | In part |
| W | 80 | Sedentary | Extreme fatigue with mild exertion and occasional fainting spells | Severe Aortic Stenosis | Atherosclerotic Heart Disease | No |
| W | 72 | Ex-smoker, sedentary, systemic arterial hypertension | Exacerbation of fatigue and palpitations with minimal exertion | Atrial Fibrillation | Systemic Arterial Hypertension | No |
| M | 61 | Smoker, diabetes mellitus 2, dyslipidemia, acute myocardial infarction | Retrosternal pain with minor exertion | Angina | Angina | Yes |
| M | 80 | Systemic arterial hypertension, dyslipidemia | Fatigue while walking with edema in the right lower extremity without dyspnea | Lipedema | Coronary Artery Disease | No |
| M | 84 | Systemic arterial hypertension, hypothyroidism, Parkinson's, atrial fibrillation | Evening edema in the left lower extremity for a week and worsening dyspnea when lying down | Deep vein thrombosis | Left Heart Failure | No |
| M | 58 | Systemic arterial hypertension, diabetes mellitus, chronic kidney disease, alcoholism | Dyspnea and chest pain, in a squeezing sensation, related to physical exertion | Myocardial Ischemia | Congestive Heart Failure | No |
| M | 49 | Systemic arterial hypertension, acute myocardial infarction | Dyspnea on moderate exertion associated with retrosternal pain and orthopnea | Acute decompensated Heart Failure | Chronic Ischemic Heart Disease | No |
| W | 63 | Diabetes mellitus 2, systemic arterial hypertension, sedentary | Dyspnea, palpitations, and precordial pain with mild exertion, associated with lower extremity edema | Acute Coronary Syndrome | Congestive Heart Failure | No |
| M | 71 | Diabetes mellitus 2, systemic arterial hypertension, stroke (2012), chronic kidney disease, smoker, ex-alcoholism | Intense dyspnea and pressure-like chest pain worsening with inspiration during minor exertion | Pulmonary Embolism | Heart Failure with Preserved Ejection Fraction | No |
| M | 72 | Systemic arterial hypertension, diabetes mellitus 2, stroke, ex-alcoholism, ex-smoker | Chest pain with a squeezing sensation radiating to the lumbar region for the past 3 days | Angina | Coronary Artery Disease | No |
| M | 65 | Acute myocardial infarction, systemic arterial hypertension, dyslipidemia | Epigastric pain, heartburn, and dyspnea worsen with moderate exertion | Gastroesophageal Reflux Disease | Angina | No |
| M | 83 | Systemic arterial hypertension, sedentary | Intermittent chest pain radiating to the left upper limb and occasional stabbing anterior chest pain during physical exertion at work | Angina | Systemic Arterial Hypertension | No |
| M | 21 | Alcoholism | Extreme fatigue | Chronic Fatigue Syndrome | Left Ventricular Hypertrophy | No |
| M | 72 | Chronic kidney disease, ex-alcoholism, sedentary, stroke | Increase in edema in the lower extremities worsening at night | Congestive Heart Failure | Chronic Renal Failure | No |
| M | 51 | Systemic arterial hypertension, congestive heart failure, chronic obstructive pulmonary disease | Dyspnea with minimal exertion, orthopnea, and paroxysmal nocturnal dyspnea | Acute decompensated Heart Failure | Congestive Heart Failure | No |
| M | 26 | Alcoholism | Episodes of syncope associated with hypotonia | Alcohol-induced Autonomic Dysfunction | Syncope | No |
| M | 87 | Coronary artery disease, dyslipidemia, systemic arterial hypertension | Dyspnea on significant exertion regressing gradually, without dyspnea in the supine position | Heart Failure | Coronary Artery Disease | No |
| M | 50 | Diabetes mellitus 2, dyslipidemia, systemic arterial hypertension | Intermittent chest pain with a tingling sensation, symptoms related to psychological stress | Microvascular Angina | Hypertensive Cardiomyopathy | No |
| M | 50 | Systemic arterial hypertension, diabetes mellitus, dyslipidemia | Dyspnea and burning chest pain on significant exertion, with sporadic tachycardias | Angina | Dilated Cardiomyopathy | No |
| W | 64 | Systemic arterial hypertension, dyslipidemia, pre-diabetes, smoker, sedentary | Temporary burning sensation in both arms every day when lying down | Reflux Esophagitis | Coronary Artery Disease | No |
| W | 78 | Systemic arterial hypertension, acute myocardial infarction (2019), dyslipidemia, sedentary | Precordial pain with minimal exertion | Angina | Coronary Artery Disease | No |
| M | 81 | Atrial fibrillation, cirrose, ex-alcoholism, sedentary | Exertional and orthopneic dyspnea, associated with edema in the lower extremities | Congestive Heart Failure | Heart Failure with Preserved Ejection Fraction | In part |
| M | 66 | Systemic arterial hypertension, diabetes mellitus, dyslipidemia, sedentary, smoker, alcoholism | Burning precordial pain on moderate exertion improves with rest | Angina | Coronary Artery Disease | No |
| M | 70 | Systemic arterial hypertension, dyslipidemia, chronic obstructive pulmonary disease, cardiomegaly, acute myocardial infarction, ex-smoker | Burning pain of intensity 3/10, particularly in the morning and evening | Gastroesophageal Reflux Disease | Coronary Artery Disease | No |
| W | 39 | Systemic arterial hypertension, sedentary | Incapacitating burning sensation in the chest and arms worsening with movement along with severe headache | Angina | Hypertensive Heart Disease | No |
| W | 41 | Systemic arterial hypertension | Persistent cough with non-blood-tinged sputum associated with dyspnea on moderate exertion | Pulmonary Embolism | Congestive Heart Failure | No |
| W | 72 | Diabetes mellitus, systemic arterial hypertension, non-alcoholic fatty liver disease, hypothyroidism | Dyspnea and precordial pain with physical exertion | Coronary Artery Disease | Angina | No |
| M | 76 | Dyslipidemia, systemic arterial hypertension, sedentary, smoker | Dyspnea with minimal exertion and edema in the lower extremities | Heart Failure | Rheumatic Heart Disease | No |
| M | 61 | Systemic arterial hypertension, diabetes mellitus, stroke (2011), sedentary | Dyspnea on exertion and swelling in the left leg, after prolonged standing, associated with an increase in temperature in the limb and paresthesia | Deep Vein Thrombosis | Heart Failure with Preserved Ejection Fraction | No |
| W | 63 | Dyslipidemia, pre-diabetes | Dyspnea and fatigue on moderate exertion associated with stabbing chest pains | Angina | Coronary Artery Disease | No |
| W | 62 | Systemic arterial hypertension, dyslipidemia, alcoholism | Chest pain with a tight sensation during significant exertion associated with palpitations | Angina | Angina | Yes |
| W | 73 | Systemic arterial hypertension, dyslipidemia, acute myocardial infarction (2013), ex-smoker | Stabbing precordial pain associated with dyspnea during higher-intensity activities | Angina | Angina | Yes |
| W | 27 | Obesity, sedentary | Headache and nuchal pain associated with nausea and vomiting | Hypertensive emergency | Secondary Hypertension | No |
| M | 69 | Systemic arterial hypertension, pre-diabetes, smoking, alcoholism | Mild precordial pain that worsens with physical exercise | Angina | Coronary Artery Disease | No |
| M | 84 | Systemic arterial hypertension, dyslipidemia, diabetes mellitus 2, ex-smoker, alcoholism, sedentary | Progressive fatigue, burning chest pain, and dyspnea | Angina | Coronary Artery Disease | No |
| W | 64 | Diabetes mellitus 2, systemic arterial hypertension, alcoholism, dyslipidemia | Low-intensity precordial pain in left lateral decubitus and dyspnea in dorsal decubitus | Acute Myocardial Infarction | Heart Failure with Preserved Ejection Fraction | No |
| M | 52 | Acute myocardial infarction (2018), systemic arterial hypertension, diabetes mellitus 2, obesity | Chest pain with a tight sensation and dyspnea on moderate exertion associated with paroxysmal nocturnal dyspnea | Heart Failure | Congestive Heart Failure | In part |
| M | 51 | Hypertension, congestive heart failure, chronic obstructive pulmonary disease, alcoholism, smoker | Dyspnea at minimal exertion, frequent dizziness, orthopnea, paroxysmal nocturnal dyspnea and precordial pain | Acute decompensated Heart Failure | Congestive Heart Failure | No |
| W | 72 | Systemic arterial hypertension, sedentary | Dyspnea and dizziness on moderate exertion | Heart Failure | Congestive Heart Failure | In part |
| W | 67 | Systemic arterial hypertension, diabetes mellitus, ex-smoker, sedentary | Dyspnea on moderate exertion without chest pain | Chronic Thromboembolic Pulmonary Hypertension | Hypertensive Cardiomyopathy | No |
| M | 78 | Systemic arterial hypertension, ex-smoker, sedentary | Dyspnea with minimal exertion, edema in the lower extremities, and chronic cough | Heart Failure | Heart Failure with Preserved Ejection Fraction | In part |
| W | 82 | Systemic arterial hypertension, ex-smoker | Burning chest pain and fatigue with moderate exertion | Angina | Aortic Stenosis with Regurgitation | No |
| M | 68 | Systemic arterial hypertension, dyslipidemia, obesity, ex-smoker, sedentary | Dyspnea on moderate exertion and left-sided chest pain | Chronic Coronary Syndrome | Chronic Obstructive Pulmonary Disease | No |
| W | 67 | Acute myocardial infarction, sedentary | Episodes of headache and nuchal pain associated with edema in the lower extremities and dry cough | Hypertensive Heart Disease | Systemic arterial Hypertension | No |
| W | 51 | Systemic arterial hypertension, dyslipidemia, sedentary | Retrosternal pain and palpitations worsen in stressful situations | Angina | Anxiety Disorder | No |
| W | 77 | Systemic arterial hypertension | Dyspnea upon waking and also on moderate exertion, with palpitations and sporadic stabbing sensations | Paroxysmal Nocturnal Dyspnea | Congestive Heart Failure | No |
| M | 56 | Systemic arterial hypertension, ex-smoker, ex-alcoholism, sedentary | Dyspnea on significant exertion and painful edema in the lower extremities | Congestive Heart Failure | Heart Failure with Preserved Ejection Fraction | In part |
| M | 80 | Systemic arterial hypertension, dyslipidemia | Dyspnea on exertion associated with palpitations | Heart Failure with Preserved Ejection Fraction | Angina | No |
| M | 86 | Stroke, systemic arterial hypertension, dyslipidemia, ex-smoker, ex-alcoholism | Dyspnea unrelated to physical exertion and precordial pain | Heart Failure | Angina | No |
| M | 89 | Diabetes mellitus, systemic arterial hypertension, sedentary | Dyspnea with minimal exertion, constant weakness in the legs, and episodes of dizziness | Heart Failure | Heart Failure with Preserved Ejection Fraction | In part |
| W | 67 | Diabetes Mellitus 2, systemic arterial hypertension, dyslipidemia, ex-smoker | Occasional stabbing chest pain on the left side and dyspnea with minor exertion | Chronic Coronary Syndrome | Angina | No |
| M | 50 | Systemic arterial hypertension, dyslipidemia, ex-smoker, alcoholism | Retrosternal pain and dyspnea during physical exertion or in situations of emotional stress | Angina | Coronary Artery Disease | No |
| W | 51 | Hypothyroidism, dyslipidemia, systemic arterial hypertension | Chest pain with a squeezing sensation radiating to the left upper limb, dyspnea on minor exertion, orthopnea, tachycardia, and edema in the lower extremities | Acute Coronary Syndrome | Congestive Heart Failure | No |
| M | 35 | Systemic arterial hypertension, dyslipidemia, sedentary | Chest pain with a squeezing sensation and spontaneously improving | Angina | Coronary Artery Disease | No |
| M | 70 | Smoker, ex-alcoholism, dyslipidemia, diabetes mellitus | Nocturnal dyspnea and cough | Acute Decompensated Heart Failure | Chronic Obstructive Pulmonary Disease | No |
| W | 59 | Ex-smoker, systemic arterial hypertension, dyslipidemia | Epigastric pain, nausea, dizziness and frequent palpitations | Gastroesophageal Reflux Disease | Systemic arterial Hypertension | No |
| M | 73 | Systemic arterial hypertension, asthma, chronic obstructive pulmonary disease, dyslipidemia, smoker | Episodes of palpitations, dizziness, and fatigue | Atrial Fibrillation | Cardiac Arrhythmia | In part |
| M | 66 | Systemic arterial hypertension, coronary artery disease, dyslipidemia | Burning precordial pain on maximal exertion and in stressful situations | Angina | Angina | Yes |
| M | 72 | Systemic arterial hypertension, dyslipidemia | Dyspnea and chest pain on moderate exertion, along with edema in the lower extremities | Heart Failure | Heart Failure | Yes |
| W | 74 | Dyslipidemia, sedentary | Fatigue upon walking with relief at rest (NYHA Class III), palpitations, and mild orthopnea | Heart Failure | Heart Failure with Preserved Ejection Fraction | In part |
| W | 46 | Sedentary | Intensifying chest pain with a tight sensation on moderate exertion, edema in lower extremities, dizziness, and dyspnea | Congestive Heart Failure | Coronary Artery Disease | No |
| W | 80 | Stroke, systemic arterial hypertension, arrhythmia, diabetes mellitus, dyslipidemia, obesity | Dyspnea on moderate exertion, orthopnea, and edema in the lower extremities | Heart Failure | Congestive Heart Failure | In part |
| W | 59 | Diabetes mellitus, dyslipidemia, sedentary | Stabbing precordial pain on moderate exertion, generalized fatigue, and palpitations at rest | Angina | Heart Failure with Preserved Ejection Fraction | No |
